# Disease-modifying therapies for relapsing/active secondary progressive multiple sclerosis – a review of population-specific evidence from randomized clinical trials

**DOI:** 10.1177/17562864221146836

**Published:** 2023-01-24

**Authors:** Antonios Bayas, Monika Christ, Simon Faissner, Juliane Klehmet, Refik Pul, Thomas Skripuletz, Sven G. Meuth

**Affiliations:** Department of Neurology, Faculty of Medicine, University of Augsburg, Augsburg, Germany; Department of Neurology, Faculty of Medicine, University of Augsburg, Augsburg, Germany; Department of Neurology, St. Josef-Hospital, Ruhr-University Bochum, Bochum, Germany; Department of Neurology, Jüdisches Krankenhaus Berlin, Berlin, Germany; Department of Neurology and Center for Translational and Behavioral Neurosciences (C-TNBS), University Medicine Essen, Essen, Germany; Department of Neurology, Hannover Medical School, Hannover, Germany; Department of Neurology, Medical Faculty, Heinrich Heine University Düsseldorf, Moorenstr. 5, Düsseldorf 40225, Nordrhein-Westfalen, Germany

**Keywords:** disease modifying treatment, multiple sclerosis, relapsing-remitting multiple sclerosis, review, secondary progressive

## Abstract

Although the understanding of secondary progressive multiple sclerosis (SPMS) is evolving, early detection of relapse-independent progression remains difficult. This is further complicated by superimposed relapses and compensatory mechanisms that allow for silent progression. The term relapsing multiple sclerosis (RMS) subsumes relapsing-remitting multiple sclerosis (RRMS) and SPMS with relapses. The latter is termed ‘active’ SPMS, for which disease-modifying therapies (DMTs) approved for either RMS or active SPMS can be used. However, the level of evidence supporting efficacy and safety in SPMS differs between drugs approved for RMS and SPMS. Our review aims to identify current evidence from published clinical trials and European public assessment reports from the marketing authorization procedure on the efficacy, especially on progression, of DMTs approved for RMS and SPMS. To identify relevant evidence, a literature search has been conducted and European public assessment reports of DMTs approved for RMS have been screened for unpublished data specific to SPMS. Only two clinical trials demonstrated a significant reduction in disability progression in SPMS study populations: the EXPAND study for siponimod, which included a typical SPMS population, and the European study for interferon (IFN)-beta 1b s.c., which included patients with very early and active SPMS. Both DMTs also achieved significant reductions in relapse rates. Ocrelizumab, cladribine, ofatumumab, and ponesimod are all approved for RMS – ocrelizumab, ofatumumab, and ponesimod based on an RMS study, cladribine based on an RRMS study. Data on efficacy in SPMS are only available from *post hoc* analyses of very small subgroups, representing only up to 15% of the total study population. For these DMTs, approval for RMS, including active SPMS, was mainly based on the assumption that the reduction in relapse rate observed in patients with RRMS can also be applied to SPMS. Based on that, the potential of these drugs to reduce relapse-independent progression remains unclear.

## Background

Multiple sclerosis (MS) is a chronic inflammatory demyelinating disease of the central nervous system associated with neurodegeneration that initially presents with a relapsing-remitting course in most patients (relapsing-remitting MS; RRMS). After a variable period, some patients develop a gradual worsening of neurological function independent of relapses, which is termed secondary progressive MS (SPMS). Therapeutic approaches in SPMS are more difficult to target due to a shift of pathogenic mechanisms involved.^1^ In this review, we will summarize clinical aspects of SPMS and the trial data of disease-modifying therapies available, specifically regarding effects on true SPMS populations.

### Definition of secondary progression

In recent years, the therapeutic landscape for SPMS and the understanding of the disease have begun to change. Nevertheless, there are no standardized objective definition criteria or biomarkers for diagnosing SPMS.^2^ In most clinical contexts, the diagnosis is made retrospectively following an increase in neurological impairment independent of relapses over 3–12 months.^3–5^ A definition of SPMS using the MSBase cohort – a large, prospectively collected, global MS cohort – proposes the following criteria (Lorscheider criteria): progression of disability independent of relapses, baseline Expanded Disability Status Scale (EDSS) ⩾ 4.0, and pyramidal Functional System Score (FSS) ⩾ 2.^4^ According to the 2014 Lublin criteria,^3^ progressive disease can be subclassified into four categories: (1) active and with progression, (2) active but without progression, (3) not active but with progression, and (4) not active and without progression (stable disease). Disease activity in this context includes clinical as well as magnetic resonance imaging (MRI) activity.^3^

Despite these definitions, clinical and paraclinical diagnostic criteria identifying early SPMS or SPMS transition are lacking.^2^ The approach by Lorscheider *et al.* is pragmatic; however, it fails to acknowledge the early signs of progression.^2,6^ These subtle signs of early progression can easily remain unnoticed due to compensating reserve capacity and superimposed relapses (‘silent progression’).^2,6^ Superimposed relapses occur more frequently in early SPMS and decrease over time.^7^ The concept of relapsing multiple sclerosis (RMS) applied by the European Medicines Agency (EMA) for regulatory purposes includes both RRMS and SPMS with relapses.^8^ Nevertheless, it remains of importance to differentiate between both disease forms. While maintaining the concept of RMS, RRMS can be considered as RMS with relapses as the main driver of disability accumulation, while SPMS with relapses can be considered as RMS with relapse-independent progression as an additional driver of disability accumulation.

In this context, a distinction between relapse-associated worsening (RAW) and progression independent of relapse activity (PIRA) has been proposed as a concept for RMS patients on higher risk of SPMS based on further analysis of the OPERA 1 and 2 studies.^9^ Deterioration is considered to be relapse-associated if it is detected within 90 days of the onset of a relapse compared with baseline and confirmed at 12 or 24 weeks. In contrast, worsening is considered to be independent of relapse activity if it occurs in comparison to a recent baseline (re-baselining) obtained no earlier than 30 days after the onset of the last relapse.^9^ Whether that distinction could be applied in a SPMS population has not been fully evaluated yet. Furthermore, limitations of this classification have been acknowledged by the authors who suggested their usage. For example, patients may not be able to recall milder relapses, and therefore these data would not be acknowledged. Furthermore, PIRA and RAW focus on relapses and do not consider focal MRI lesions.^9^ This limits their value for detection of early signs of SPMS, as both relapses and focal lesions are correlates of the peripheral inflammatory processes that drive progression in active disease.^10^

According to the EMA, it is accepted that approvals for RMS mainly relies on the effects shown in patients with RRMS and that an effect on relapses in RRMS may be extrapolated to an effect on relapses in SPMS.^8^ Nevertheless, the increasing role of relapse-independent disease progression in SPMS requires distinguishing between RMS and SPMS to evaluate and further develop disease-modifying therapies (DMTs) that are effective in early SPMS patients.

### Treatment recommendations for active SPMS

For decades, mitoxantrone and interferon (IFN)-beta 1b have been the only available treatments for SPMS. During this time period, the therapeutic landscape has changed dramatically, and new DMTs have emerged for RMS or active SPMS based on their clinical efficacy data in either RMS or SPMS studies, including ocrelizumab, ofatumumab, cladribine, ponesimod, and siponimod.^11–15^ Other DMTs have failed to demonstrate efficacy in SPMS patients in SPMS-specific studies (IFN-beta 1a i.m., IFN-beta 1a s.c., and natalizumab) or have had insufficient data on patients representative for SPMS from (R)RMS studies (dimethyl fumarate, teriflunomide, peginterferon beta-1a s.c., and ozanimod).^16–21^

Clinical practice guidelines of the American Academy of Neurology (AAN) published in 2018 do not include specific DMT recommendations for SPMS, but highlight that patients with active SPMS, either relapses or MRI lesions, benefit from DMTs.^22^ Guidelines published by the European Committee for Treatment and Research in Multiple Sclerosis (ECTRIMS) in 2018 provided a weak recommendation to consider IFN-beta 1a s.c. or IFN-beta 1b s.c., mitoxantrone, ocrelizumab, or cladribine for patients with active SPMS.^23^ The 2021 update of the ECTRIMS guideline has not yet been published at the time of this review. Most current recommendations are available from the German Neurological Society (Deutsche Gesellschaft für Neurologie, DGN), last updated in 2021. In addition to the 2018 ECTRIMS recommendations, the DGN guideline further includes siponimod for active SPMS. On the contrary, mitoxantrone, despite its history as an SPMS medication, is only recommended as reserve medication for RMS and after other therapeutic options have been exhausted.^24^ According to the DGN guideline, young age, short duration of disease, low degree of disability, superimposed relapses, or rapid increase in disability, and evidence of inflammatory activity on MRI support DMT use in SPMS.^24^ Available DMTs are not recommended for patients with inactive or non-relapsing SPMS, but in untreated patients with inactive SPMS, rapid increase in disability and impending loss of independence, a therapeutic attempt, initially limited to 2 years, with an anti-CD20 antibody (like in primary progressive MS), can be considered in individual cases. The lack of evidence and the risks of therapy should be discussed in detail with the patient.^24^

### Approval status of DMTs recommended for active SPMS

The SPMS treatment recommendations include DMTs approved for active SPMS or RMS (Table 1). The RMS label allows the prescription of DMTs for SPMS with relapses. However, as outlined before, the RMS indication mainly relies on RRMS relapse data that has been extrapolated to active SPMS.^8^

**Table 1.** Approval status of DMTs used for active SPMS / RMS treatment.

Drug	SPMS/RMS-relevant EU label	SPMS/RMS-relevant US label
Cladribine (Mavenclad)^25,26^	Indicated for the treatment of adult patients with highly active relapsing multiple sclerosis (MS) as defined by clinical or imaging features.	Indicated for the treatment of relapsing forms of multiple sclerosis (RMS), to include relapsing-remitting disease and active secondary progressive disease, in adults.
Dimethyl fumarate (Tecfidera)^27^	None	Indicated for the treatment of patients with RMS
Diroximel fumarate (Vumerity)^28^	None	Indicated for RMS, to include clinically isolated syndrome, relapsing-remitting disease, and active secondary progressive disease, in adults.
IFN-beta 1a i.m. (Avonex)^29^	None	For the treatment of RMS, to include clinically isolated syndrome, relapsing-remitting disease, and active secondary progressive disease, in adults.
IFN-beta 1a s.c. (Rebif)^30,31^	Indicated for the treatment of (…) relapsing MS. In clinical trials, this was characterized by two or more acute exacerbations in the previous 2 years. Efficacy has not been demonstrated in patients with secondary progressive MS without ongoing relapse activity.	Indicated for the treatment of patients with RMS to decrease the frequency of clinical exacerbations and delay the accumulation of physical disability.
IFN-beta 1b s.c. (Betaferon/Betaseron, Extavia)^32,33^	Indicated for the treatment of (…) patients with secondary progressive MS with active disease, evidenced by relapses.	Indicated for the treatment of RMS, to include clinically isolated syndrome, relapsing-remitting disease, and active secondary progressive disease, in adults.
Mitoxantrone (Novantrone, Ralenova)^34,35^	Indicated for treatment of patients with highly active relapsing MS associated with rapidly evolving disability, where no alternative therapeutic options exist.	Indicated for reducing neurologic disability and/or the frequency of clinical relapses in patients with secondary (chronic) progressive, progressive relapsing, or worsening relapsing-remitting MS (i.e., patients whose neurologic status is significantly abnormal between relapses).
Natalizumab (Tysabri)^36^	None	Indicated for the treatment of patients with RMS to delay the accumulation of physical disability and reduce the frequency of clinical exacerbations; generally recommended for patients who have had an inadequate response to, or are unable to tolerate, an alternate MS therapy.
Ocrelizumab (Ocrevus)^37,38^	Indicated for the treatment of adult patients with RMS with active disease defined by clinical or imaging features; (in addition: early primary progressive MS with inflammatory activity).	RMS, to include clinically isolated syndrome, relapsing-remitting disease, and active secondary progressive disease, in adults; (in addition: primary progressive MS, in adults).
Ofatumumab (Kesimpta)^39,40^	Indicated for the treatment of adult patients with RMS with active disease defined by clinical or imaging features.	Indicated for the treatment of RMS, to include clinically isolated syndrome, relapsing-remitting disease, and active secondary progressive disease, in adults.
Ozanimod (Zeposia)^41^	None	Indicated for the treatment of RMS, to include clinically isolated syndrome, relapsing-remitting disease, and active secondary progressive disease, in adults.
Peginterferon beta-1a s.c. (Plegridy)^42^	None	Indicated for the treatment of RMS, to include clinically isolated syndrome, relapsing-remitting disease, and active secondary progressive disease, in adults.
Ponesimod (Ponvory)^43,44^	Indicated for the treatment of adult patients with RMS with active disease defined by clinical or imaging features.	Indicated for the treatment of RMS, to include clinically isolated syndrome, relapsing-remitting disease, and active secondary progressive disease, in adults.
Siponimod (Mayzent)^45,46^	Indicated for the treatment of adult patients with secondary progressive multiple sclerosis (SPMS) with active disease evidenced by relapses or imaging features of inflammatory activity^a^.	Indicated for the treatment of RMS, to include clinically isolated syndrome, relapsing-remitting disease, and active secondary progressive disease, in adults.
Teriflunomide (Aubagio)^47^	None	Indicated for the treatment of patients with RMS.

a Gd-enhancing T1 lesions or new/enlarging T2 lesions.

Mitoxantrone had originally been approved for SPMS in several European countries, including Germany, but the license was changed to RMS with rapidly evolving disability in 2016 during an EU harmonization process.^48^ In the United States, mitoxantrone is still indicated for SPMS. IFN-beta 1a s.c. is approved for RMS in both Europe and the United States, whereas IFN-beta 1b s.c. is approved for SPMS with relapse activity in Europe and for RMS in the United States. Ocrelizumab, ofatumumab, cladribine, and ponesimod are approved for RMS in both Europe and the United States. Siponimod is specifically approved for SPMS with active disease in Europe and for RMS in the United States and for SPMS in some other countries. IFN-beta 1a i.m., peginterferon beta-1a, natalizumab, teriflunomide, dimethyl fumarate, diroximel fumarate, and ozanimod have no SPMS or RMS marketing authorization in Europe, whereas they have US approval for RMS (Table 1).

**Figure 1.**
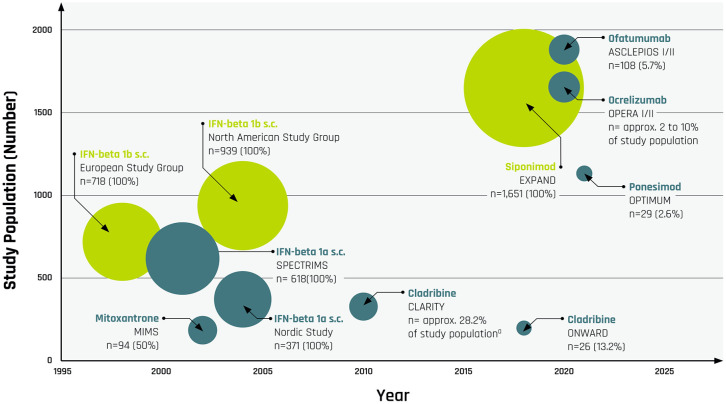
Size of SPMS subgroups relative to total study population in clinical trials for DMTs approved for active SPMS or RMS by year of RCT publication; y-axis denotes the size of the entire study population, x-axis denotes the year of RCT publication; bullet size denotes the relative size of the SPMS subgroup; *n* = total number of SPMS patients (percentage of SPMS patients in the total study population); yellow bullets denote SPMS label; blue bullets denote RMS label. ^a^Cladribine: 374 patients (28.2%) had a baseline EDSS of 4 or higher, indicating that a proportion of patients may have transitioned to SPMS.

It has to be kept in mind that the level of evidence with respect to efficacy in SPMS differs between RMS- and SPMS-approved medications. Specifically, the latter is based on evidence from SPMS-specific study populations, while RMS labels are based on RMS study populations with only small SPMS subgroups (Figure 1). SPMS-focused evidence concerning efficacy of DMTs recommended for SPMS treatment and approved for use in SPMS or RMS will be reviewed in the following sections.

### SPMS-specific evidence from randomized trials

#### Literature search

Embase, Medline, and Biosis have been searched in December 2021 to identify randomized controlled trials (RCTs) with DMTs in active SPMS. Furthermore, European public assessment reports (EPARs) from the marketing authorization procedure of DMTs approved for RMS and SPMS have been screened for data relating specifically to SPMS. EPARs were searched and accessed *via* the website of the European Medicines Agency (EMA).

#### Mitoxantrone and beta-interferons

Historically, mitoxantrone and beta-interferons were the only drugs available for SPMS patients. However, the clinical evidence regarding beta-interferons available in this population was heterogeneous, especially with respect to disability progression. Mitoxantrone is cytotoxic and induces DNA damage. It inhibits B- and T-cell proliferation and the excretion of inflammatory cytokines.^49^ Beta-interferons belong to the regulatory cytokines and exert pleiotropic effects on the immune system, including a shift to anti-inflammatory cytokine signaling.^50^ Both mitoxantrone and beta-interferons do not cross the blood–brain barrier and thus do not exert direct central effects.

Clinical efficacy of mitoxantrone in SPMS was investigated in the mitoxantrone in secondary progressive multiple sclerosis (MIMS) study. Here, both SPMS patients and patients with progressive RMS were included in a 1:1 ratio. In the total study population, mitoxantrone was significantly superior to placebo in terms of reduction in relapse risk and frequency, as well as 3-month confirmed disability progression (CDP) and 6-month CDP. There was also a significant reduction in lesion burden.^51^ Patients in the MIMS study were characterized by pronounced relapse activity.^23^ Thus, the MIMS study included a population of active SPMS patients eligible for therapy.

Four RCTs assessed the efficacy of IFN-beta 1a s.c. (SPECTRIMS, Nordic Study) and IFN-beta 1b s.c. (European Study, North American Study) in SPMS. One trial investigated IFN-beta 1a i.m., which is not approved for use in SPMS (IMPACT) (Table 2). A Cochrane meta-analysis demonstrated that beta-interferons were not effective in delaying disability progression in SPMS patients. Data on 6-month CDP was available for three studies (Nordic Study, European Study, and North American Study). Accordingly, 6-month CDP occurred in 41.0% of patients treated with placebo and in 38.3% of patients treated with beta-interferons over 3 years (*p* = 0.79). A small but statistically significant reduction in the risk of relapse was found.^52^ Subgroup analyses indicated that efficacy was primarily observed in the subgroup of patients with superimposed relapse activity.^52^

**Table 2.** RCT with data on DMT efficacy in active SPMS or RMS including SPMS patients.

DMT	EU-Label for active SPMS / RMS	US Label for active SPMS / RMS	Clinical trial with data on SPMS/RMS, initial publication year	Comparator	Study population	SPMS-specific population, *n* (%)	Data for SPMS available	Primary endpoint met
Cladribine	Highly active RMS	RMS	CLARITY 2010^13,53^	Placebo	RRMS, *N* = 1,326	374 (28.2)^a^	No	Yes
			ONWARD 2018^13,54^ (add-on to IFN-beta)	Placebo	RMS, *N* = 197^b^	26 (13.2) SPMS with relapses	From *post hoc* subgroup analysis	Yes
IFN-beta 1a i.m.	None	RMS	IMPACT 2002^16^	Placebo	SPMS, *N* = 436	436 (100)	Yes	No
IFN-beta 1a s.c.	RMS	RMS	SPECTRIMS 2001^21,55^	Placebo	SPMS, *N* = 618	618 (100)	Yes	No
			Nordic Study 2004^56^	Placebo	SPMS, *N* = 371	371 (100)	Yes	No
IFN-beta 1b s.c.	SPMS with relapses	RMS	European Study Group 1998^57–59^	Placebo	SPMS, *N* = 718	718 (100)	Yes	Yes
			North American Study Group 2004^58,60^	Placebo	SPMS, *N* = 939	939 (100)	Yes	No
Mitoxantrone	Highly active RMS	SPMS	MIMS 2002^51^	Placebo	SPMS / progressive RRMS, *N* = 188^c^	94 (50.0)	No	Yes
Natalizumab	None	RMS	ASCEND 2018^17^	Placebo	SPMS, *N* = 889	889 (100)	Yes	No
Ocrelizumab	Active RMS	RMS	OPERA I/II 2017^9,11,61^	IFN-beta 1a	RMS, *N* = 1,656	1.9% to 10.2%	From *post hoc* subgroup analysis	Yes
Ofatumumab	Active RMS	RMS	ASCLEPIOS I/II 2020^12,62^	Teriflunomide	RMS, *N* = 1,882	108 (5.7) active SPMS	From *post hoc* subgroup analysis	Yes
Ponesimod	Active RMS	RMS	OPTIMUM 2021^15,63^	Teriflunomide	RMS, *N* = 1,133	29 (2.6) SPMS with relapses	From *post hoc* subgroup analysis	Yes
Siponimod	Active SPMS	RMS	EXPAND 2018^64^	Placebo	SPMS, *N* = 1,651	1.651 (100)	Yes	Yes
Teriflunomide	None	RMS	TOWER 2014^65^	Placebo	RMS, *N* = 1,169	9 (0.8)	No	Yes
			TEMSO 2011^66^	Placebo	RMS, *N* = 1,088	51 (4.7)	No	Yes
			TENERE 2014^67^	IFN-beta 1a	RMS, *N* = 324	1 (0.3)	No	No

IFN, interferon; i.m., intramuscular; *N*, number of randomized patients; RMS, relapsing multiple sclerosis; RRMS, relapsing-remitting multiple sclerosis; s.c., subcutaneous; SPMS, secondary progressive multiple sclerosis.

a A total of 374 (28%) had a baseline EDSS of 4 or higher, indicating that a proportion of patients may have transitioned to SPMS.

b The original protocol included two doses of cladribine (3.5 mg/kg and 5.25 mg/kg). By protocol amendment, the higher dose of cladribine was omitted. In total, 197 patients were included in the placebo and cladribine 3.5 mg/kg groups of the original (*N* = 25) and the amended protocol (*N* = 172).

c A total of 194 patients were randomized. Of these, 188 received treatment and had at least one efficacy assessment. These patients were included in the analysis. MS subtype is available for the analysis population only.

A meta-analysis on IFN-beta 1b s.c. by Nikfar *et al.*^68^, including the European Study and the North American Study, determined a relative risk (RR) for relapse of 0.93 [95% confidence interval (CI) [0.75, 1.14]] compared with placebo. When the Nordic Study for IFN-beta 1a s.c. data were included, the relapse RR was 1.11 (95% CI [0.79, 1.55]), indicating no significant effect in relapse prevention compared with placebo.^68^ No data are available from this meta-analysis regarding CDP.

The pivotal study on peginterferon beta-1a (ADVANCE) only included RRMS patients. In this population, peginterferon beta-1a effectively reduced the relapse rate compared with placebo.^69^ During the marketing authorization procedure, the applicant argued that one in five patients of the study population could be considered to be similar to SPMS patients. However, no subgroup data on these patients are available.^70^

#### Ocrelizumab

Ocrelizumab is a monoclonal antibody directed against CD20+ cells in the periphery. CD20 is highly expressed on the cell surface of B-lineage cells and is widely considered a B-cell-specific marker. However, CD20 is also expressed on a small subset of CD3+ T cells.^71^ Recent data show that treatment with ocrelizumab does not exclusively affect B cells, but also CD20+ T cells.^72^ Ocrelizumab is not known to directly target the central nervous system.^73^ While the ORATORIO phase 3 clinical trial demonstrated reduced risk of disability progression of ocrelizumab in primary progressive MS with a hazard ratio of 0.75 (95% CI [0.58, 0.98]; *p* = 0.04) at week 24,^74^ no SPMS-specific studies have been performed for ocrelizumab. The randomized and double-blind phase 3 OPERA I and II studies compared ocrelizumab with IFN-beta 1a s.c. and included 821 (OPERA I) and 835 (OPERA II) patients with RMS (Table 2). In the entire RMS population, the studies demonstrated a reduction in annualized relapse rate (ARR), 12-week and 24-week CDP, gadolinium (Gd)-enhancing T1 lesion load as well as new or enlarging T2 lesion load and Multiple Sclerosis Functional Composite Scores (MSFC, in OPERA 2 only) by ocrelizumab compared with IFN-beta 1a s.c.^61^

RMS subtype was not documented at baseline.^11^ However, *post hoc* analyses attempted to identify potential SPMS patients by applying different definitions. Accordingly, as published by Kappos *et al.*,^9^ 21.4% of patients had an increased likelihood of SPMS defined as baseline EDSS ⩾ 4.0 and pyramidal FSS ⩾ 2. In an additional approach described in the EPAR, potential SPMS patients had been identified through re-baselining for EDSS, Timed 25-Foot Walk (T25FW), and 9-hole peg test (9-HPT) for each patient after each relapse and the requirement of subsequent progression in the absence of relapse. Furthermore, the effect of treatment was estimated in a subgroup identified using the Lorscheider criteria, that is, disability progression independent of relapse, baseline EDSS ⩾ 4.0, and pyramidal FSS ⩾ 2.^4,11^ The EPAR states that with these approaches, 1.9–10.2% of the intention-to-treat population were identified as possible SPMS patients. It is not specified to what this range refers. In addition, no information is given on the extent of relapse-independent CDP and the extent of relapse activity in this subpopulation at baseline.^11^

According to the EPAR, ocrelizumab has a superior benefit compared with IFN-beta 1a s.c. on relapse-independent disability progression in the SPMS subpopulation.^11^ In detail, the analysis identified a 24% risk reduction in relapse-independent 12-week composite CDP (*p* = 0.0098) and a 22% risk reduction in relapse-independent 24-week composite CDP (*p* = 0.0456) (Table 3).

**Table 3.** SPMS-specific results of DMTs from RCTs.

Intervention (Clinical trial)^a^	*N* (SPMS)	Comparator	*N* (SPMS)	% of study	ARR^a^	Disability Progression^a^	MRI^a^
Cladribine (+ IFN-beta) (ONWARD)^13,54^	17	Placebo (+ IFN-beta)	9	13.2	RR 0.11; 95% CI [0.01, 0.94]; *p* = 0.0439	3 m-CDP HR 1.1; 95% CI [0.28, 4.42]; *p* = n.a. 6 m-CDP HR 0.78; 95% CI [0.13, 4.67] *p* = n.a.	Gd^+^ T1 lesions (mean, SD) 0.13 ± 0.55 *versus* 0.67 ± 2.00; *p* = n.a. Active T2 lesions (mean, SD) 0.29 ± 0.52 *versus* 0.59 ± 1.66; *p* = n.a.
IFN-beta 1a i.m. (IMPACT)^16^	217	Placebo	219	100	ARR reduction 33%; *p* = 0.008	3 m-CDP HR 0.977; 95% CI [0.679, 1.407]; *p* = 0.90	Gd^+^ T1 lesions (proportion of patients with Gd^+^ lesions) 13% *versus* 28%; *p* < 0.001 New/enlarging T2 lesions (reduction of mean number) 45.6% (month 24); *p* < 0.001
IFN-beta 1a s.c. (SPECTRIMS)^21,55^	204^b^	Placebo	205	100	Rate ratio 0.69; 95% CI [0.56, 0.85]; *p* < 0.001	3 m-CDP HR 0.83; 95% CI [0.65, 1.07]; *p* = 0.146	Active T2 lesions per patient/scan (median; Q1, Q3) 0.17 (0, 0.50) *versus* 0.67 (0.17, 2.00); *p* < 0.001
IFN-beta 1a s.c. (Nordic Study)^56^	186^c^	Placebo	178	100	Rate ratio 0.90; 95% CI [0.64, 1.27]; *p* = 0.55	6 m-CDP HR 1.13; 95% CI [0.82, 1.57]; *p* = 0.45	n.a.
IFN-beta 1b s.c. (European study group)^57–59^	360	Placebo	358	100	ARR reduction 33%; *p* = 0.0001	6 m-CDP HR 0.70; 95% CI [0.55, 0.88]; *p* = n.a.	Gd^+^ T1 lesions (reduction of mean number) 67%; *p* = n.a. T2 lesion load (median % change) −6.91 *versus* 2.96; *p* = n.a.
IFN-beta 1b s.c. (North American Study Group)^58,60^	317^d^	Placebo	308	100	ARR reduction 43%; *p* = 0.0091	6 m-CDP HR 0.93; 95% CI [0.71, 1.22]; *p* = n.a.	Gd^+^ T1 lesions (reduction of mean number) 83%; *p* = n.a. T2 lesion load (median % change) 0.42 *versus* 10.9; *p* = n.a.
Natalizumab (ASCEND)^17^	439^e^	Placebo	448	100	Rate ratio 0.453; 95% CI [0.323, 0.634]; *p* < 0.001	6 m-CDP OR 0.86; 95% CI [0.66, 1.13]; *p* = 0.287	Gd^+^ T1 lesions (mean change, SD) −0.93 ± 5.01 *versus* 0.10 ± 4.12; *p* < 0.001 New/enlarging T2 lesions (mean change, SD) 0.8 ± 3.67 *versus* 7.1 ± 13.03; *p* < 0.001
Ocrelizumab (OPERA I + II)^9,11,61^	n.a.	IFN-beta 1a s.c.	n.a.	1.9–10.2	n.a.	12w-CDP risk reduction 24%; *p* = 0.0098 24w-CDP risk reduction 22%; *p* = 0.0456	n.a.
Ofatumumab (ASCLEPIOS I + II)^12,62^	56	Teriflunomide	52	5.7	Rate ratio 0.57; 95% CI [0.23, 1.38]; *p* = n.a.	6 m-CDP risk reduction 44%; *p* = 0.228	n.a.
Ponesimod (OPTIMUM)^15,63^	15	Teriflunomide	14	2.6	Rate ratio 1.299; 95% CI [0.538, 3.134]; *p* = n.a.	Time to first 12w-CDA HR: 0.69; 95% CI [0.16, 2.87]; *p* = n.a.	Cumulative number of CUAL Rate ratio: 0.088; 95% CI [0.020, 0.386]; *p* = n.a.
Siponimod (EXPAND)^64^	1,099^f^	Placebo	546	100	Rate ratio 0.45; 95% CI [0.34, 0.59]; *p* < 0.0001	3 m-CDP HR 0.79; 95% CI [0.65, 0.95]; *p* = 0.013 6 m-CDP HR 0.74; 95% CI [0.60, 0.92]; *p* = 0.0058	Gd^+^ T1 lesions Rate ratio 0.14; 95% CI [0.10, 0.19]; *p* < 0.0001 New or enlarging T2 lesions Rate ratio 0.19; 95% CI [0.16, 0.24]; *p* < 0.0001

ARR, annual relapse rate; CDA, confirmed disability accumulation; CDP, confirmed disability progression; CI, confidence interval; CUAL, combined unique active lesions; Gd, gadolinium; HR, hazard ratio; IFN, interferon; i.m., intramuscular; m, month; MRI, magnetic resonance imaging; N, number of randomized patients; n.a., not available; ns, not significant; OR, odds ratio; Q1, 25% quartile; Q3, 75% quartile; RR, risk ratio; s.c., subcutaneous; SD, standard deviation; SPMS, secondary progressive multiple sclerosis; w, week.

a Results are presented as intervention *versus* comparator.

b Results for the approved standard dose (44 µg) are presented.

c Study investigated IFN-beta 1a s.c. 22 µg; 371 patients were randomized. Of these, 364 received treatment and were included in the analysis.

d Results for the approved standard dose (250 µg) are presented.

e A total of 889 patients were randomized, 887 were included in the intention-to-treat population.

f A total of 1105 patients were randomized to the siponimod arm, but 6 were excluded from efficacy analysis.

Based on these *post hoc* analyses, the Committee for Medicinal Products for Human Use (CHMP) acknowledged that ocrelizumab consistently reduces the risk of progression across all assessed measures of disability, including relapse-independent disability.^11^ However, the CHMP also stated that it cannot be excluded that the effect of ocrelizumab on disability may rather be driven by its effect on inflammation and on inflammation-related disability accumulation than by effects on neurodegeneration-related disability.^11^

#### Ofatumumab

Ofatumumab is a CD20-targeting antibody, and, like ocrelizumab, it is not known to exert direct central nervous system effects.^73^ No SPMS-specific studies have been performed for ofatumumab. Efficacy and safety have been assessed in the randomized, double-blind, ASCLEPIOS I and II trials.^62^ Overall, 946 RMS patients were assigned to ofatumumab and 936 to teriflunomide (Table 2). In the total RMS population, the ARR was significantly reduced by ofatumumab compared with teriflunomide in both trials. A pooled analysis showed significant reduction in 3-month and 6-month confirmed disability worsening by ofatumumab *versus* teriflunomide. Gd-enhancing lesions and new or enlarging T2 lesions were also significantly reduced by ofatumumab in both trials. Data regarding brain volume loss indicated a trend for improvement under ofatumumab treatment.^62^

In total, 108 patients (5.7%) of the ASCLEPIOS study populations had active SPMS.^62^ SPMS-specific results from a pooled analysis revealed that ofatumumab reduced the ARR by 43% (rate ratio: 0.57; 95% CI [0.23, 1.38]) and the risk of 6-month CDP by 44% (*p* = 0.228) (Table 3). The CHMP concluded that these results should be interpreted with caution as the SPMS group was very small, and the confidence interval was broad. The CHMP further stated that efficacy regarding relapses in RRMS patients may be extrapolated to SPMS, but extrapolation on disability progression was deemed inappropriate due to differences in the underlying pathophysiology.^12^

#### Cladribine

Cladribine is a nucleoside analogue of deoxyadenosine. It belongs to the class of antimetabolites.^53^ It can cross the blood–brain barrier; however, no MS-specific targets have thus far been identified. For cladribine, no trial specifically in SPMS has been performed. The placebo-controlled CLARITY trial^53^ included patients with a previous diagnosis of RRMS who had experienced a relapse in the previous year. Approximately 28.2% of the participants had a baseline EDSS ⩾ 4, indicating that a relevant proportion might have already transitioned to SPMS (Table 2).^53^ In this study, cladribine significantly reduced ARR compared with placebo, the 3-month sustained progression of disability, and MRI lesion count. No efficacy results have been published for the CLARITY subpopulation of patients with baseline EDSS ⩾ 4.^53^

The randomized ONWARD trial evaluated the additional use of oral cladribine in combination with IFN-beta 1a s.c. in patients with relapse activity despite beta-interferon therapy.^54^ The original protocol included two doses of cladribine (3.5 mg/kg and 5.25 mg/kg). By protocol amendment, the higher dose of cladribine was omitted. In total, 197 patients were included in the placebo and cladribine 3.5 mg/kg groups of the original (*N* = 25) and the amended protocol (*N* = 172) (Table 2). Cladribine in addition to IFN-beta 1a s.c. significantly reduced relapse risk by 63% compared with IFN-beta 1a s.c. plus placebo in the amended protocol study population. No effect of cladribine was observed regarding disability progression but lesion burden was reduced.^54^ In total, 26 patients with active SPMS (13.2%) were included in the ONWARD study. In the SPMS subgroup, cladribine plus IFN-beta 1a s.c. demonstrated an 89% reduction in the ARR compared with placebo (RR 0.11; 95% CI [0.01, 0.94]). No effect of cladribine was observed in the SPMS subgroup with respect to time to 3-month CDP [hazard ratio (HR) 1.1; 95% CI [0.28, 4.42]] or 6-month CDP (HR 0.78; 95% CI [0.13, 4.67]). Both the number of Gd-enhancing T1 lesions (0.13 ± 0.55 *versus* 0.67 ± 2.00; mean ± SD) and the number of active T2 lesions (0.29 ± 0.52 *versus* 0.59 ± 1.66; mean ± SD) were reduced by cladribine compared with placebo plus IFN-beta (Table 3).^54^

A pooled analysis of patient subpopulations from ONWARD and CLARITY using baseline EDSS ⩾ 3.5 as a proxy for SPMS or high risk of transitioning to SPMS is available from the EPAR. Accordingly, the ARR risk ratio was 0.47 in the EDSS ⩾ 3.5 subgroup.^13^ The CHMP concluded that based on the results from CLARITY and ONWARD, and to maintain consistency with other approved MS DMTs, efficacy regarding relapses in RRMS patients may be extrapolated to SPMS. The CHMP was of the view that the appropriate target population for cladribine would be patients with highly active RMS instead of RRMS, for which a license was initially applied.^13^

#### Siponimod

Siponimod is a second-generation oral sphingosine-1-phosphate (S1P) receptor modulator with selectivity for receptor subtypes S1PR1 and S1PR5. In contrast to the aforementioned DMTs, siponimod effectively crosses the blood–brain barrier and enters the central nervous system (CNS) where it causes direct effects on astrocytes, microglia, oligodendrocytes, and neurons in animal models mediated through S1PR.^75^ In addition to the reduction in focal inflammatory disease activity, siponimod directly manipulates CNS intrinsic inflammatory processes relevant to SPMS on activated microglial cells and macrophages.^75,76^ It is postulated that these effects are not solely dependent on S1PR1-directed activity, but also involve S1PR5. S1PR5 is expressed in the CNS on oligodendrocytes and their progenitor cells and might be involved in the modulation of myelin repair and oligodendrocyte survival. Direct CNS effects *via* S1PR5 may play a critical role in the effectiveness in SPMS.^76,77^

For siponimod, large-scale SPMS-specific data are available. Efficacy and safety of siponimod were assessed in the randomized, double-blind, placebo controlled EXPAND study.^64^ This study was specifically designed to investigate efficacy in an SPMS population with typical characteristics such as high level of disability (>50% using walking aid at study entry) and low levels of inflammatory activity. It therefore delivers robust results on siponimod use in this population, specifically. Overall, 1,651 SPMS patients with evidence of disability progression in the previous 2 years were included in the study (Table 2). Participants represent a typical SPMS population in terms of age (mean 48 years), SPMS duration (mean 4 years), relapse (64% relapse-free), and MRI activity (Gd^+^ lesions in 21%), and baseline EDSS ⩾ 6 in 55.6% of the population (Table 4). In comparison, age and SPMS duration in the European Study and the North American interferon beta-1b study was rather similar. However, relapse activity was by far lower in the European Study and slightly lower in the North American Study, while MRI activity was slightly higher in the European Study and by far higher in the North American Study (Table 4).

**Table 4.** Patient characteristics in SPMS trials.

Parameter	IFN-beta 1b s.c. (European Study group)^57–59^		IFN-beta 1b s.c. (North American Study Group)^58,60^		Siponimod (EXPAND)^64^	
	IFN-beta 1b s.c. *N* = 360	Placebo *N* = 358	IFN-beta 1b s.c^a^ *N* = 317	Placebo *N* = 308	Siponimod *N* = 1105^b^	Placebo *N* = 546
Age in years, mean (SE/SD)	41.1 (SD 7.2)	40.9 (SD 7.2)	46.1 (SE 0.45)	47.6 (SE 0.46)	48.0 (SD 7.8)	48.1 (SD 7.9)
Female, *n* (%)	209 (58.1)	230 (64.2)	210 (66.2)	185 (60.1)	669 (60.5)	323 (59.2)
Duration of MS since diagnosis in years, mean (SE/SD)	8.1 (SD 5.6)	8.2 (SD 6.1)	14.6 (SE 0.44)	14.9 (SE 0.48)	12.9 (SD 7.9)	12.1 (SD 7.5)
Duration of SPMS in years, mean (SE/SD)	3.8 (SD 2.7)	3.8 (SD 3.4)	4.0 (SE 0.19)	4.1 (SE 0.20)	3.9 (SD 3.6)	3.6 (SD 3.3)
EDSS, mean (SE/SD)	5.1 (SD 1.1)	5.2 (SD 1.1)	5.2 (SE 0.06)	5.1 (SE 0.07)	5.4 (SD 1.1)	5.4 (SD 1.0)
EDSS ⩾ 6, *n* (%)	153 (42.5)	169 (47.2)	n.a.	n.a.	622 (56.3)	296 (54.2)
Relapse-free in prior 2 years, *n* (%)	115 (31.9)	101 (28.2)	170 (53.6)	174 (56.5)	712 (64.4)^c^	343 (62.8)^c^
Proportion of patients with Gd^+^ T1 lesions, %	30		55		21	

Gd, gadolinium; IFN, interferon; MRI, magnetic resonance imaging; N, number of randomized patients; n.a., not available; s.c., subcutaneous; SD, standard deviation; SE, standard error; SPMS, secondary progressive multiple sclerosis; w, week.

a Only results for the approved standard dose (250 µg) are presented.

b A total of 1105 patients were randomized to the siponimod arm, but 6 were excluded from efficacy analysis.

c For three patients in the siponimod and one patient in the placebo group, information on the number of relapses in the past 2 years was not available.

Siponimod significantly reduced the risk of disability progression by 21% (3-month CDP: HR 0.79; 95% CI [0.65, 0.95]; *p* = 0.013) and 26% (6-month CDP: HR 0.74; 95% CI [0.60, 0.92]; *p* = 0.0058) (Table 3).^64^ In the subgroup of patients with active SPMS (47.2%), defined as having relapses in the 2 years before inclusion or with evidence of Gd-enhancing T1 lesions on baseline MRI, the delay in disability progression was also more pronounced. This subpopulation also showed typical features of SPMS similar to the overall population regarding EDSS, disease duration, or age, and only differed in terms of having evidence of recent disease activity. In this active SPMS population, 3-month CDP was reduced by 31% (*p* = 0.0094) and 6-month CDP by 37% (*p* = 0.0040) *versus* placebo.^78^ Clinical benefits on disability progression were observed in patients with active SPMS irrespective of age.^79^ However, in the overall SPMS population, the effect on disability progression was more pronounced in patients aged up to 40 years compared with older patients and in patients with a duration of SPMS of up to 10 years compared with a longer disease duration.^64^

Risk of 6-month CDP based on the MSIS-29 physical score with a clinically meaningful cutoff of 7.5 also decreased in the overall population (HR 0.78, *p* = 0.0147), in the active SPMS population (HR 0.73, *p* = 0.030), and in patients aged ⩽ 45 years (HR 0.63, *p* = 0.005). The same applies to 6-month CDP based on the 12-item Multiple Sclerosis Walking Scale (MSWS-12) with clinically meaningful cutoffs of 6, 8, and 10 points in the overall population (HR 0.75–0.80, *p* < 0.05), the active SPMS subgroup (HR 0.72–0.74, *p* < 0.05) and patients aged ⩽ 45 years (HR 0.67–0.71, *p* < 0.05).^80^ No significant benefit was observed with respect to T25FW, which might be due to low sensitivity of the T25FW in patients who are already severely impaired regarding their walking performance.

According to a *post hoc* analysis from the EXPAND study, siponimod reduced the risk of sustained, clinically meaningful worsening in cognitive processing speed (⩾4 points on the Symbol Digit Modalities Test, SDMT) by 28% compared with placebo (*p* = 0.0166) in patients with active SPMS. It improved the chance of sustained improvement in cognitive processing speed by 51% (*p* = 0.0070).^78^

The ARR was reduced by 55% compared with placebo (rate ratio 0.45; 95% CI [0.34, 0.59]; *p* < 0.001) in the overall population.^64^ The cumulative number of Gd-enhancing T1 lesions per scan (rate ratio 0.14; 95% CI [0.10, 0.19]; *p* < 0.0001) as well as the mean number of new or enlarging T2 lesions (rate ratio 0.19; 95% CI [0.16, 0.24]; *p* < 0.0001) was significantly reduced.^64^

Cree *et al.*^81^ determined the extent to which the effects on 3- and 6-month CDP were relapse-independent. The authors estimated the effect based on three statistical approaches. Risk reductions independent of relapse were 14–20% and 23–33% for 3- and 6-month CDP in non-relapsing patients, respectively. The analyses support the use of siponimod in SPMS patients irrespective of relapse activity.

In a matching-adjusted indirect treatment comparison of DMTs in SPMS, siponimod was significantly more effective than IFN-beta 1a and IFN-beta 1b at reducing the CDP risk. With respect to ARR, siponimod was numerically but not statistically superior to all comparators, except for natalizumab.^82^

Overall, EXPAND demonstrated that siponimod addresses acute inflammatory disease activity in the form of relapses, Gd-enhancing T1 and new or enlarging T2 lesions, as well as chronic disease progression. Evidence from EXPAND supports the use of siponimod in a typical SPMS population with an even greater benefit in early or active SPMS. Based on this more pronounced effect, the CHMP issued its positive opinion toward the use of siponimod in SPMS patients with active disease, defined by relapses or MRI activity.^14^

#### Ponesimod

Ponesimod is a S1P modulator with specificity for S1PR1.^83^ It lacks activity against S1PR5, which is thought to be relevant for direct central effects, as outlined before. The efficacy of ponesimod was evaluated in the phase 3 study OPTIMUM, a randomized, double-blind study with RMS patients (RRMS or SPMS with relapses).^63^ In total, 1133 patients were randomized to either ponesimod (*N* = 567) or teriflunomide (*N* = 566) (Table 2). In the RMS population, ponesimod significantly reduced ARR. No significant effect was observed with respect to confirmed disability accumulation (CDA). Regarding MRI endpoints, ponesimod significantly reduced the cumulative number of combined unique active lesions (CUAL).^63^

Only 3% of the study population were SPMS patients (Table 3). No significant effects for ARR (rate ratio: 1.299; 95% CI [0.538, 3.134]), 12-week CDA (HR: 0.69; 95% CI [0.16, 2.87]), and patient-reported fatigue (FSIQ-RMS mean difference: −11.87; 95% CI [−28.87, 5.13]) were observed in the SPMS subpopulation.^15^ However, a significant reduction in the cumulative number of CUALs by ponesimod was observed (rate ratio: 0.088; 95% CI [0.020, 0.386]). The CHMP approved its use as an RMS indication based on extrapolating the RRMS results on relapse risk reduction to SPMS.^15^

### Further DMTs

Natalizumab is a humanized monoclonal antibody against alpha 4-integrin. Its efficacy in SPMS has been assessed in the randomized, double-blind ASCEND study.^17^ In total, 889 patients with typical SPMS characteristics very similar to the EXPAND study population were randomized to natalizumab or placebo (Table 2). No significant effect was observed with respect to CDP assessed by EDSS, T25FW, and 9HPT (OR 0.86; 95% CI [0.66, 1.13]; *p* = 0.287) (Table 3). Regarding the single components, only 9HPT progression was significantly reduced by natalizumab (OR 0.56; 95% CI [0.40, 0.80]; *p* = 0.001).^17^

Ozanimod is an S1P modulator with specificity for S1P1 and S1P5. Although ozanimod has the same receptor specificity as siponimod, both differ in in several aspects, such as molecule structure, receptor affinity, or metabolization.^83^ The pivotal studies RADIANCE and SUNBEAM recruited RMS patients; however, no SPMS patients were included in the ozanimod 1 mg study group, which is the final approved dose (0.92 mg) of ozanimod. Therefore, no relevant SPMS-specific data are available from either study. A pooled analysis of both studies (Table 5)^18,19^ revealed no significant effect on disability progression.

**Table 5.** Results on disability progression from pivotal trials of DMTs approved for RMS by FDA only.

Intervention (Clinical trial)	N	Comparator	*N*	Population	Disability progression^a^
Dimethyl fumarate^b,20^ (DEFINE^84^ /	410^c^	Placebo	408	RRMS	3 m-CDP in RRMS patients HR 0.62; 95% CI [0.44, 0.87]; *p* = 0.005 6 m-CDP in RRMS patients HR 0.77; 95% CI [0.52, 1.14]; *p* = 0.1893
CONFIRM)^85^	359^c^	Placebo^d^	363	RRMS	3 m-CDP in RRMS patients HR 0.79; 95% CI [0.52, 1.19]; *p* = 0.2536 6 m-CDP in RRMS patients HR 0.67; 95% CI [0.40, 1.11]; *p* = 0.1172
Ozanimod (pooled analysis of RADIANCE and SUNBEAM)^86^	880^e^	IFN-beta 1a i.m.	889	RRMS	3 m-CDP in RRMS patients HR 0.950; 95% CI [0.679, 1.330]; *p* = 0.7651 6 m-CDP in RRMS patients HR 1.413; 95% CI [0.922, 2.165]; *p* = 0.1126
Peginterferon beta 1a (ADVANCE)^69^	512^f^	Placebo	500	RRMS	3 m-CDP in RRMS patients HR 0.62; 95% CI [0.40, 0.97]; *p* = 0.0383
Teriflunomide (pooled analysis of TOWER and TEMSO)^65,87^	728^g^	Placebo	751	RMS	3 m-CDP in RMS study population HR 0.695; 95% CI [0.542, 0.892]; *p* = 0.0029 6 m-CDP in RMS study population HR 0.759; 95% CI [0.570, 1.011]; *p* = 0.055 3 m-CDP in subgroup of patients with SPMS or progressive RMS^h^ HR 0.553; 95% CI [0.166, 1.839]; *p* = n.a.

CDP, confirmed disability progression; CI, confidence interval; HR, hazard ratio; m, month; *N*, number of randomized patients; n.a., not available; RMS, relapsing multiple sclerosis; RRMS, relapsing-remitting multiple sclerosis; SPMS, secondary progressive multiple sclerosis; w, week.

a Results are presented as intervention *versus* comparator.

b For diroximel fumarate, no pivotal efficacy study was available, as efficacy was extrapolated from dimethyl fumarate based on pharmacokinetic studies.

c Results for the approved dose (240 mg twice daily) are presented.

d The active comparator arm of the study (glatiramer acetate) is not presented, no significant effect on disability progression.

e Results for the approved dose (1 mg) are presented.

f Results for the approved dose (125 µg every two weeks) are presented.

g Results for the standard dose in adults (14 mg) are presented.

h Teriflunomide 14 mg: *N* = 30; placebo: *N* = 44.

Dimethyl fumarate among others activates the nuclear factor (erythroid-derived 2)-like 2 antioxidant pathway and thereby inhibits neuroinflammation. At therapeutic doses, diroximel fumarate and dimethyl fumarate produce bioequivalent systemic exposure of monomethyl fumarate.^88^ The Phase 3 studies DEFINE and CONFIRM evaluating dimethyl fumarate only included RRMS patients.^84,85^ Results on disability progression in the RRMS population of DEFINE and CONFIRM are inconsistent. In the DEFINE study, a significant reduction in CDP was observed compared with placebo, but not in the CONFIRM study (Table 5). The CHMP concluded that subgroup data of patients defined as being representative of SMPS by the applicant were insufficient to allow for extrapolation to an RMS therapeutic indication.^20^ The marketing authorization for diroximel fumarate is mainly based on pharmacokinetic bridging between dimethyl fumarate and diroximel fumarate supplemented by safety data.^89^

Teriflunomide is a reversible inhibitor of dihydroorotate dehydrogenase, which is involved in pyrimidine synthesis for DNA replication. In the placebo-controlled teriflunomide studies TEMSO and TOWER, as well as in the TENERE study using IFN-beta 1a s.c. as comparator, the number of SPMS patients was very limited.^65–67^ The TENERE study failed to meet the combined primary endpoint (treatment failure defined by relapse or treatment discontinuation) in the total study population.^67^ While TEMSO and TOWER successfully proved efficacy of teriflunomide in the total study population, a pooled subgroup analysis of patients with SPMS and progressive RMS did not show a benefit of teriflunomide over placebo regarding relapse rates (RR: 1.086; 95% CI [0.531, 2.221]). Disability progression was only numerically reduced by teriflunomide compared to placebo in the SPMS and progressive RMS subgroup (Table 5). The CHMP concluded that efficacy data were insufficient to be extrapolated to a RMS population.^87^

## Conclusions and interpretation of SPMS data

Only two DMT studies with SPMS-specific study populations successfully demonstrated benefit in terms of reduction of disability progression. These were the EXPAND study for siponimod in a typical SPMS population and the European Study for IFN-beta 1b s.c. in very early and active SPMS patients. Both, in addition, proved to be efficacious in preventing relapses.^64,57^ The North American Study for IFN-beta 1b s.c. was conducted with a SPMS population at a later disease stage compared with the European Study. In comparison to the EXPAND study, the population of the North American Study was rather similar except for a higher MRI activity. However, the North American Study did not demonstrate any benefit regarding disability progression.^60^ Among these study populations, patients from EXPAND most closely correspond to the SPMS population in clinical practice in terms of disease activity, as according to data from a German SPMS registry,^90^ 30.9% of SPMS patients have active and 69.1% have inactive disease. The average age of SPMS patients in clinical practice is 56 years and duration of SPMS since conversion is 6 years, which means that patients in the EXPAND study are slightly younger and have shorter disease duration.

Further DMT recommendations for SPMS treatment are based on RMS studies with very small SPMS subpopulations. Marketing authorization for RMS including SPMS with relapses is mainly based on the extrapolation of efficacy data in terms of relapse rate reduction seen in patients with RRMS to patients with active SPMS.^8^ This implies that evidence for these DMTs is vague compared with evidence on DMTs generated from real SPMS studies. Furthermore, published results from *post hoc* analyses of these SPMS subpopulation in RMS trials in most cases do not allow for estimate of the extent of relapse-dependent and relapse-independent progression at baseline. For these DMTs, it remains unclear which subgroup of SPMS patients benefit the most, making informed treatment decisions at least difficult if not impossible.

Considering that the conduction of SPMS studies is feasible, as EXPAND and ASCEND show, these should be strongly preferred over RMS studies for the evaluation of SPMS treatments. SPMS-specific studies have the power to detect treatment effects in features relevant to SPMS other than superimposed relapses, like relapse-independent progression or cognitive decline. So far, SPMS subgroups of RMS studies are not powered to identify treatment differences in these parameters and thus do not generate interpretable results for SPMS patients. Although clinical safety was not a focus of this review, it should be at least noted that RMS studies are neither powered to identify treatment-induced risks in elderly patients, nor do SPMS subgroup data from RMS studies adequately inform treatment decisions in SPMS patients from a safety point of view.

Despite active SPMS and RRMS being subsumed under the term RMS and despite sharing overlapping features, they differ greatly. As outlined before, RRMS can be considered as RMS with relapses as the main driver of disability accumulation, whereas SPMS with relapses can be considered as RMS with relapse-independent progression as an additional relevant driver of disability accumulation. It can be assumed that new RMS therapies suppress relapse activity in SPMS and thus at least partially mitigate disability progression. However, clear evidence on this is lacking due to the small sizes of SPMS subgroups in most RMS studies. Moreover, the relevance of relapse activity decreases over time, and relapse prevention can be assumed to be less important in SPMS patients in the long term. Relapse-independent progression becomes increasingly important in this population, but it is subject to different pathological mechanisms and CDP data from RRMS patients cannot be extrapolated to SPMS. This has also been highlighted in the EMA guidelines.^8^ Although the concept of RMS increases the therapeutic armamentarium for early and relapsing SPMS, this should not obscure the need for appropriately powered studies and for drugs specifically approved for SPMS.

Despite the observed effects of siponimod on relapse-independent disability progression, siponimod data underscore the importance of early therapy. Treatment effects were particularly pronounced in younger patients and patients with shorter disease duration. On the contrary, age did not have an effect in patients with active SPMS. This suggests that the treatment effect is greater when inflammatory activity is present. Lorscheider *et al.* have analyzed the potential effect of anti-inflammatory DMTs on disability outcomes in SPMS from MSBase data. Of the 2381 included patients, 689 treated and 689 untreated patients were matched. Differences between matched treated and untreated patients were neither observed for 6-month CDP (HR 0.9; 95% CI [0.7, 1.1]; *p* = 0.27), nor for the risk of reaching a confirmed EDSS ⩾ 7 (HR 0.6; 95% CI [0.4, 1.1]; *p* = 0.10). The authors suggested that anti-inflammatory DMTs have no substantial effect on relapse-independent disability progression in SPMS without a distinct inflammatory phenotype.^91^ However, these results are based on registry data and should be interpreted with caution.

The extent to which underlying inflammatory processes might have impacted the results of SPMS studies remains uncertain. Future study designs must take this into account and apply measures to distinguish between inflammatory and non-inflammatory related progression. The concepts of PIRA and RAW are heading in this direction; however, using relapses as sole correlates of peripheral inflammation. Inclusion of MRI activity into these measures might increase the relevance of PIRA and RAW for future studies. Studies addressing currently discussed aspects of SPMS pathogenesis, that is, intrinsic inflammatory processes,^92^ oxidative stress, mitochondrial damage, and other mechanisms driving neurodegeneration^1^ are needed. These should include assessments of slowly expanding lesions including cortical lesions as well as cortical atrophy, which are more common in progressive MS.^93,94^ In addition, serum neurofilaments as well as glial fibrillary acidic protein are currently discussed as potential biomarkers in SPMS.^95,96^ Optical coherence tomography (OCT) is increasingly used in MS studies to assess neurodegeneration and might be indicative of progressive MS, suggesting OCT as a potentially suitable tool in SPMS trials.^97^ Moreover, more suitable clinical outcomes are needed, for example, using composite scores which assess gait and upper limb function using T25FW and 9HPT.^98^ Furthermore, it would be helpful to have a uniform definition of ‘progression’ as a study endpoint addressing all relevant aspects in SPMS. Mostly, studies are still designed using EDSS-based disability progression, supplemented by measures like T25FW or 9HPT. Accordingly, the EXPAND study used EDSS-based 3-month CDP and confirmed worsening of at least 20% in the T25FW to define disability progression, which in the light of the study results seems quite feasible for a SPMS-specific study.^64^ However, a recent analysis on the reliability of outcome measures like EDSS, T25FW, and 9HPT showed some evidence of random variation and measurement error.^99^ This at least questions the usability of these measures for the detection of subtle signs of progression. This not only applies to progression as an endpoint, but also to progression in terms of SPMS diagnosis and, therefore, study population selection. The identification of more suitable measures and definitions for progression is urgently needed in SPMS. They are a prerequisite for the development of DMTs that effectively delay progression independently from peripheral inflammation and that are potentially effective even in late SPMS.

## Declarations

**Ethics approval and consent to participate:** Not applicable.

**Consent for publication:** Not applicable.

**Availability of data and materials:** Not applicable.
